# Synthesis and biological evaluation of the novel chrysin prodrug for non-alcoholic fatty liver disease treatment

**DOI:** 10.3389/fphar.2024.1336232

**Published:** 2024-04-19

**Authors:** Ruiming Zhang, Chuanyue Gao, Mingxing Hu, Xingxing Wang, Shuoyuan Li, Zhenmei An, Xifei Yang, Yongmei Xie

**Affiliations:** ^1^ Department of Nuclear Medicine, State Key Laboratory of Biotherapy and Cancer Center, West China Hospital, Sichuan University and Collaborative Innovation Center of Biotherapy, Chengdu, China; ^2^ Shenzhen Key Laboratory of Modern Toxicology, Shenzhen Medical Key Discipline of Health Toxicology (2020–2024), Shenzhen Center for Disease Control and Prevention, Shenzhen, China; ^3^ Department of Endocrinology and Metabolism, West China Hospital, Sichuan University, Chengdu, China

**Keywords:** non-alcoholic fatty liver disease (NAFLD), chrysin, prodrug, pharmacokinetic property, PPAR signaling pathway

## Abstract

**Background:** Chrysin (5,7-dihydroxyflavone) is a natural flavonoid that has been reported as a potential treatment for non-alcoholic fatty liver disease (NAFLD). However, extensive phase II metabolism and poor aqueous solubility led to a decrease in the chrysin concentration in the blood after oral administration, limiting its pharmacological development *in vivo*.

**Methods:** In the present study, we synthesized a novel chrysin derivative prodrug (C-1) to address this issue. We introduced a hydrophilic prodrug group at the 7-position hydroxyl group, which is prone to phase II metabolism, to improve water solubility and mask the metabolic site. Further, we evaluated the ameliorative effects of C-1 on NAFLD *in vitro* and *in vivo* by NAFLD model cells and db/db mice.

**Results:** *In vitro* studies indicated that C-1 has the ability to ameliorate lipid accumulation, cellular damage, and oxidative stress in NAFLD model cells. *In vivo* experiments showed that oral administration of C-1 at a high dose (69.3 mg/kg) effectively ameliorated hyperlipidemia and liver injury and reduced body weight and liver weight in db/db mice, in addition to alleviating insulin resistance. Proteomic analysis showed that C-1 altered the protein expression profile in the liver and particularly improved the expression of proteins associated with catabolism and metabolism. Furthermore, in our preliminary pharmacokinetic study, C-1 showed favorable pharmacokinetic properties and significantly improved the oral bioavailability of chrysin.

**Conclusion:** Our data demonstrated that C-1 may be a promising agent for NAFLD therapy.

## 1 Introduction

Non-alcoholic fatty liver disease (NAFLD) is a chronic liver disease associated with several metabolic syndromes (Hardy et al., 2016). In recent years, the prevalence of NAFLD has shown a gradual increase globally because of changes in people’s lifestyles and dietary structures (Riazi et al., 2022). NAFLD is characterized by excessive accumulation of hepatic lipids and insulin resistance and may be associated with metabolism-related disorders such as obesity, hyperglycemia, and hypertriglyceridemia (Fujii et al., 2020). Approximately 59.10% of patients with NAFLD have been reported to progress to non-alcoholic steatohepatitis, with hepatic steatosis, an inflammatory response, and cellular damage within 5 years, which may eventually lead to cirrhosis, hepatocellular carcinoma, and end-stage liver failure (Younossi et al., 2019). Unfortunately, there are no clinically effective interventions for NAFLD beyond strict dietary control and regular exercise (Mantovani and Dalbeni, 2021). Therefore, there is an urgent need to discover an efficient drug to treat NAFLD.

**FIGURE 1:**
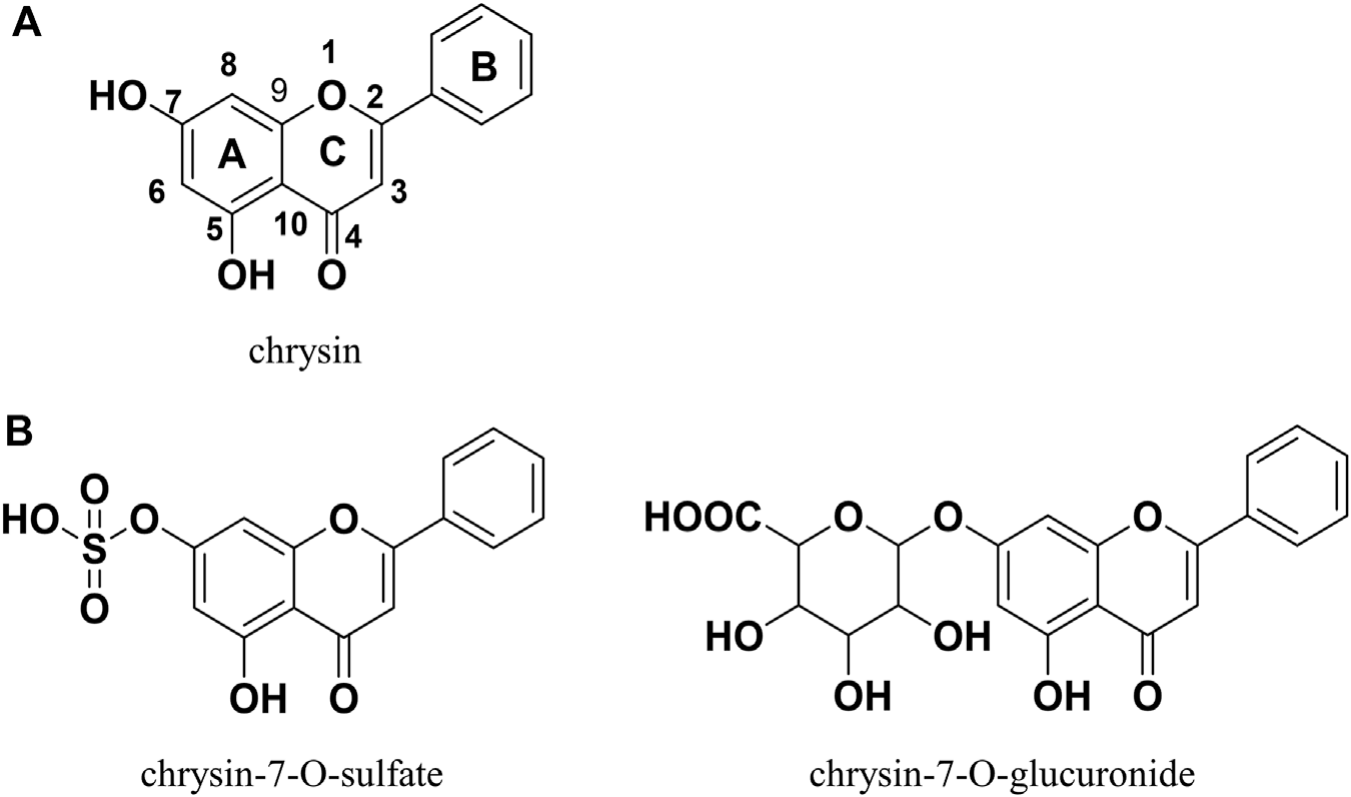
Chrysin and its two dominant products of metabolism in humans.

In recent years, natural products have received increasing attention for their limited side effects, low cost, and suitability for long-term use (Baek et al., 2021; Li et al., 2023). Chrysin (Figure 1A) is a natural flavonoid with a wide range of biological activities that is widely present in honey, propolis, fruits, and various medicinal plants (Mani and Natesan, 2018). Many reports in the literature describe the potential of chrysin for the treatment of NAFLD, demonstrating its ability to ameliorate hepatic inflammation, hepatic fat accumulation, altered glucolipid metabolism, and insulin resistance (Pai et al., 2019; Song et al., 2020; Zhou et al., 2021; Liu et al., 2023; Ye et al., 2023). However, the oral bioavailability of chrysin is very low because of its poor water solubility and rapid metabolism in the gastrointestinal tract and liver. A pharmacokinetic study in human volunteers showed that more than 90% of chrysin is excreted as aglycone in the feces, with the remainder excreted in the urine as chrysin or chrysin-glucuronide; the estimated oral bioavailability was less than 1% (Walle et al., 2001). This finding may explain why chrysin performs well *in vitro* and not as well *in vivo*. Therefore, chrysin needs to be chemically modified to improve its bioavailability, metabolic stability, and consequently, its *in vivo* bioactivity.

The prodrug approach is a common chemical modification strategy for active natural compounds aimed at optimizing pharmacodynamic and pharmacokinetic properties by modulating physicochemical properties including solubility, chemical or enzymatic stability, bioavailability, and toxicity (Abet et al., 2017). The carbamate structure has been used in the design of a variety of active compound prodrugs, such as SN38 (Sadzuka et al., 2005), resveratrol (Mattarei et al., 2015), quercetin (Kim et al., 2009), and baicalein (Son et al., 2021). This structure is commonly used to protect the highly reactive phenolic hydroxyl group to form the -OXR structure, where X is a carbamate and R is a group that improves physicochemical properties and is non-toxic. This protective linkage is reversible, helping to circumvent the effects of phase II metabolism and releasing active compounds (Mattarei et al., 2015). *In vitro* and *in vivo* studies have shown that chrysin, similar to other naturally occurring polyphenols, generates mainly phase II metabolism products (e.g., chrysin-7-O-glucuronide and chrysin-7-O-sulfate; Figure 1B) mediated by UDP-glucuronosyltransferases and sulfotransferases during absorption, with the 7-O position being the major metabolic site for this process (Stompor-Gorący et al., 2021). Appropriate chemical protection of the 7-O position using the carbamate structure helps to improve the physicochemical properties of chrysin and enhance its metabolic stability and biological activity.

In the present study, we synthesized a novel chrysin-carbamate derivative, C-1, and further investigated its potential therapeutic mechanism against NAFLD *in vitro* and *in vivo*. In addition, proteomic analyses revealed differential protein expression after C-1 treatment, and GO and KEGG analysis demonstrated the main functions and possible pathways of action of the differential proteins. Pharmacokinetic experiments in rats were also performed to evaluate the *in vivo* absorption, stability, and metabolism of the new prodrug.

## 2 Materials and methods

### 2.1 Chemistry and characterization

#### 2.1.1 Reagents

Chrysin and 1-chlorocarbonyl-4-piperidinopiperidine hydrochloride were obtained from Chengdu Biotech Chemical Technology Ltd. (Sichuan, China). Reagents used in the synthesis of C-1 were all purchased from commercial suppliers and used without purification. All solvents were dried according to standard methods before use. Dimethyl sulfoxide and CCK-8 were obtained from Sigma-Aldrich (St Louis, MO, United States). Column chromatography was carried out using a silica gel (200–300 mesh, Sichuan Kelong Chemical Ltd., Sichuan, China). Thin-layer chromatography (TLC) was performed on TLC silica gel 60 F254 plates. ^1^H NMR and ^13^C NMR spectra were measured on a Bruker AV-400 spectrometer (400 MHz). ^1^H NMR spectrometry and chemical shifts (δ) were reported in parts per million relative to tetramethylsilane, which was used as an internal standard.

#### 2.1.2 Synthesis of compound C-1

**FIGURE 2:**
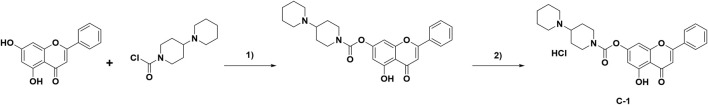
Synthesis of compound C-1. Reagents and conditions: 1) K_2_CO_3_, acetonitrile, 75 °C, 8 h. 2) 2 mol/L HCl in 1,4-dioxane solution, rt, 2 h.

Chrysin (254 mg, 1.0 mmol) was dissolved in a round-bottom flask in CH_3_CN (20 mL). Potassium carbonate (483 mg, 3.5 mmol) and 1-chlorocarbonyl-4-piperidinopiperidine hydrochloride (267 mg, 1.0 mmol) were added to the above reaction system, and the reaction was refluxed for 8 h. When the reaction was completed, the organics were concentrated under reduced pressure. After water (30 mL) was added to the flask, the organic layer was extracted with dichloromethane (30 mL × 3), dried with sodium sulfate, and then filtered and concentrated under reduced pressure. Finally, the residue was purified by preparative TLC, mixed with a solution of 2 mol/L HCl in 1,4- dioxane (15 mL), and stirred at room temperature for 2 h. The solvent was then concentrated under reduced pressure and dried to produce C-1 (400 mg) with an 82.5% yield. The route of C-1 synthesis is shown in Figure 2.

#### 2.1.3 Detection of C-1 solubility

C-1 was dissolved in 10 mL of water (pH 7.4) to prepare a specific concentration of the control solution. Additionally, C-1 was dissolved in water to form a supersaturated solution, which was centrifuged, passed through a membrane, and diluted by pipetting a small amount of the solution. Subsequently, the absorption peak areas of the two solutions at the maximum absorption wavelength were determined separately by HPLC, and the concentration of the measured solution was calculated according to the following equation:

$$C_{X}{=(A}_{X}{/A}_{0}{)\;C}_{0}$$

C_X_ is the concentration of the measured solution; A_X_ is the area of the absorption peak of the measured solution; C_0_ is the concentration of the control solution; and A_0_ is the area of the absorption peak of the control solution.

### 2.2 Biology

#### 2.2.1 Cell culture and treatment

The human hepatic LO2 cell line was purchased from the American Type Culture Collection (ATCC, Rockville, MD, United States). Cells were propagated in Dulbecco’s modified Eagle’s medium containing 10% fetal bovine serum (HyClone, Logan, UT, United States) and 1% antibiotics (penicillin and streptomycin) in 5% CO_2_ at 37 °C. When the confluence reached 60%–70%, LO2 cells were induced with 100 μM sodium oleate for 24 h to produce lipid deposition. Then, LO2 cells were treated with 10 μM or 20 μM chrysin or C-1 for 24 h. Thereafter, the biochemical indices of the cells were measured. The condition of LO2 cell differentiation and the ameliorative effect of C-1 after sodium oleate treatment were confirmed by detecting the total cholesterol (TC), triglyceride (TG), malondialdehyde (MDA), superoxide dismutase (SOD), aspartate aminotransferase (AST), and alanine aminotransferase (ALT) levels.

#### 2.2.2 Cell viability

LO2 cells were inoculated in 96-well plates at a density of 3,000–5,000 cells/well for 24 h. Cell viability was analyzed in cells treated with various concentrations of sodium oleate (0, 0.2, 0.4, 0.6, 0.8, and 1.0 mM), chrysin, and C-1 (0, 5, 10, 20, and 40 μM for both) for 24 h using a CCK-8 assay. The optical density value was read at 450 nm using a microplate reader.

#### 2.2.3 Oil red O staining

LO2 cells (5 × 10^5^) were seeded in six-well plates and treated with 0.2 mM sodium oleate for 24 h, followed by treatment with different concentrations of chrysin and C-1 for 24 h. Staining of the above cells was carried out using an oil red O staining kit (Beijing Solarbio Science & Technology Co., Ltd. Beijing, China). Lipid droplets in the cells were observed and imaged using a light microscope.

#### 2.2.4 Detection of TC, TG, MDA, and SOD in cells

The cells were collected and lysed. The TC, TG, MDA, and SOD levels in cells were detected using commercial reagent kits (Solarbio Science & Technology Co., Ltd.).

#### 2.2.5 Detection of AST and ALT activity in cell culture medium supernatants

The medium was collected after culturing the cells, and the supernatant was centrifuged. AST and ALT activities were measured using commercial kits (Jiancheng Institute of Biotechnology, Nanjing, China) according to the manufacturer’s instructions.

#### 2.2.6 Animals and treatment

Eight-week-old male diabetic C57BLKS/J (db/db) mice (n = 18) and their age-matched controls (dbm, n = 6) were purchased from Changzhou Cavens Laboratory Animal Co., Ltd. (Jiangsu, China). Mice were placed in cages at a stable temperature (23°C–25 °C) and humidity with a 12-h light/dark cycle from 7:00 a.m. to 7:00 p.m.

The dbm mice were used as the control group. All db/db mice were randomly divided into a model group, a C-1 high-dose treatment group (HC-1), and a C-1 low-dose treatment group (LC-1) (six mice per group). The mice in the control and model groups were treated with saline (20 mL/kg/d) by gavage, and the HC-1 and LC-1 groups were treated with C-1 (69.3 mg/kg/d or 23.1 mg/kg/d, respectively) by gavage for 8 weeks; the body weights of the mice were recorded once per week. At the end of the treatment cycle, the mice were sacrificed after being fasted for 12 h and anesthetized with pentobarbital sodium. Blood samples were collected in anticoagulation tubes (Sinopharm Shanghai Pharmaceutical Co., Ltd. Shanghai, China) and centrifuged at 4 °C and 10,000 × g for 5 min to obtain the upper plasma layer. Liver tissues were quickly removed, weighed, and divided into two parts; one part was fixed in 4% paraformaldehyde for histopathological examination, and the other was stored at −80 °C for further biochemical assays. Subsequently, various biochemical indices in mouse serum were assayed using commercial reagent kits.

#### 2.2.7 Ethical statement

Animal experiments were approved by the Institutional Animal Care and Treatment Committee of the State Key Laboratory of Biotherapy at Sichuan University.

#### 2.2.8 Serum biochemical analysis

The TG, TC, low-density lipoprotein cholesterol (LDL-C), and high-density lipoprotein cholesterol (HDL-C) levels in the serum were detected using commercial reagent kits from Solarbio Science & Technology Co. The AST, ALT, alkaline phosphatase (ALP), α-hydroxybutyrate dehydrogenase (HBDH), and lactate dehydrogenase (LDH) levels in the serum were detected using commercial reagent kits from Jiancheng Institute of Biotechnology.

#### 2.2.9 Tissue sections and staining

Liver tissues were immersed in formalin for more than 24 h, embedded in paraffin, and sectioned into 3-μm thin sections using a microtome. The sections were stained with hematoxylin-eosin or periodic acid-Schiff and then observed under a microscope at ×200 magnification.

#### 2.2.10 Evaluation of the acute toxicities of C-1

Experiments were conducted by our outsourcing company, Shanghai Medicilon Biomedical Co Ltd. (Shanghai, China), in accordance with the National Drug Administration (NMPA) “Technical Guidelines for Toxicity Studies of Drugs in Single Administration” (May 2014), following the generally accepted procedures for the detection of pharmaceutical compounds. Briefly, 24 healthy SD rats (SPF grade), half male and half female, were selected and divided into four groups by simple randomization. Each group consisted of three animals of each sex. Group 1 was a solvent control group at a dose of 0 mg/kg body weight; groups 2–4 were C-1 administration groups at doses of 500, 1,000, and 2000 mg/kg body weight, respectively. All groups were administered one dose by oral gavage. At the end of the experiment (Day 8), all surviving animals in groups 1–4 were euthanized by CO_2_ inhalation anesthesia.

### 2.3 Preliminary pharmacokinetic studies

#### 2.3.1 Animals

Twelve SPF grade adult male SD rats, 10–12 weeks old, weighing 200 ± 20 g, were supplied by Shanghai Medicilon Biomedical Co Ltd. The rats were given free access to water and a normal diet. Favorable feeding conditions (light/dark cycle 12/12 h, temperature 25°C ± 1 °C, humidity 55% ± 5% and pathogen-free environment) were provided. The animals were fasted overnight (10–14 h) before drug administration, and food was given 4 h after drug administration.

#### 2.3.2 Drug administration and sample collection

The animals were divided into four groups of three rats each, which were administered chrysin (50.0 mg/kg p.o. and 10.0 mg/kg i.v.) and C-1 (95.4 mg/kg p.o. and 19.1 mg/kg i.v.), for which the dose of C-1 was obtained by equimolar conversion of the dose of chrysin. Blood samples were collected through the jugular vein after drug administration at several preset time points, including 0.25 h, 0.5 h, 1 h, 2 h, 4 h, 6 h, 8 h, and 24 h for the p.o. group and 0.083 h, 0.25 h, 0.5 h, 1 h, 2 h, 4 h, 8 h, and 24 h for the i.v. group. Each sample was approximately 0.2 mL, and the blood was placed in a heparinized EP tube. After centrifugation for 6 min at 4 °C, 6,800 rpm, the supernatant was removed to obtain blank plasma, which was stored at −80 °C.

#### 2.3.3 Treatment of plasma samples

A 20-µL plasma sample aliquot was protein-precipitated with 200 µL of MeOH that contained 100 ng/mL IS (tolbutamide for the analysis of C-1, warfarin for the analysis of chrysin). The mixture was vortexed for 1 min and centrifuged at 18,000 × g for 7 min. The supernatant (200 µL) was transferred to a 96-well plate. A 6-µL aliquot of supernatant was injected for liquid chromatography-tandem mass spectrometry (LC-MS/MS) analysis.

#### 2.3.4 LC-MS/MS conditions

The samples were analyzed using an LC-MS/MS system (SCIEX Triple Quad™ 6,500+, MA, United States). The chromatographic conditions were as follows. A Luna^®^ Omega 1.6-µm Polar C18 100 50*2.1 mm column was used. The mobile phase consisted of 0.1% trifluoroacetic acid in H_2_O (A) - 0.1% trifluoroacetic acid in MeCN (B), the flow rate was 0.6 mL/min, the detection wavelength was 254 nm, the column temperature was 40 °C, and the injection volume was 6 μL. The elution procedure was as follows: 0.01–0.60 min, 10%–90% (B), 0.60–1.10 min, 90%–90% (B), 1.10–1.11 min, 90%–10% (B), 1.11–1.40 min, 10%–10% (B) for the analysis of C-1; 0.01–0.60 min, 10%–50% (B), 0.60–0.80 min, 50%–50% (B), 0.80–1.20 min, 50%–90% (B), 1.20–1.60 min, 90%–90% (B), 1.60–1.61 min, 90%–10% (B), 1.61–1.80 min, 10%–10% (B) for the analysis of chrysin.

### 2.4 Proteomic analysis

#### 2.4.1 Sample preparation

Urea (8 M) was added to the liver tissue samples, which were then completely lysed using an ultrasonic homogenizer and kept on ice for 30 min. The lysate was centrifuged at 12,000 × g, 4 °C for 30 min to obtain protein solution. The protein concentration was detected using a commercial kit (Thermo Fisher Scientific, NJ, United States).

The sample was labeled according to a previously reported method (Xu et al., 2019). Each sample, containing 100 µg of protein, was digested with mass spectrometric trypsin (Promega, V5072) into peptides and then labeled with tandem mass tag (TMT) 6-plex reagents (Thermo Scientific). The labeled peptides from each sample were then mixed, desalted, dried, and finally dissolved in 100 μL of 0.1% formic acid. Subsequently, the TMT-labeled samples were divided into 15 components using HPLC for subsequent experiments.

#### 2.4.2 LC-MS/MS analysis and database searches

The dried ingredients were dissolved in 0.1% FA and captured on a silica capillary column packed with C18 resin (Varian, MA, United States) for subsequent Q Extractive (Thermo Scientific) mass spectrometry analysis. Proteome Discoverer 2.5 software (Thermo Scientific) was used to retrieve MS/MS data according to the reviewed uniport-mouse database and in reference to previous study settings (Xu et al., 2019).

#### 2.4.3 Bioinformatics analysis

The data were analyzed using Maxquant statistical software. Proteins with a value of *p* < 0.05 were evaluated using a *t*-test and considered differentially expressed. Hiplot software was used for functional enrichment analysis of Gene Ontology for biological processes and molecular function and pathway analysis.

### 2.5 Statistical analysis

Data were represented as mean ± standard deviation (S.D.) of three or more independent experiments. Data were analyzed using one-way ANOVA followed by Tukey’s test. Differences were considered statistically significant at *p* < 0.05.

## 3 Results

### 3.1 Synthesis and structural characterization

A prodrug strategy is commonly used for modification of natural products to improve their water solubility and metabolic stability and reduce toxicity. Structure-activity relationship studies of existing novel chrysin derivatives have shown that modification of the 7-O position of chrysin improves its biological activity and pharmacokinetic properties (Müller-Schiffmann et al., 2010; Singh et al., 2015). Using the synthesis method described above (Figure 2), 400 mg of C-1 was obtained with a yield of 82.5%. The structures were characterized by ^1^H NMR, ^13^C NMR, and MS, and the spectra are provided in the Supplementary Figure S1–S3. The data are as follows: ^1^H NMR (400 MHz, CDCl_3_) δ 12.72 (s, 1H), 7.91–7.83 (m, 2H), 7.60–7.48 (m, 3H), 6.89 (d, *J* = 2.0 Hz, 1H), 6.72 (s, 1H), 6.58 (d, *J* = 2.0 Hz, 1H), 4.52–4.41 (m, 2H), 3.60–3.46 (m, 2H), 3.40–3.28 (m, 1H), 3.16–3.04 (m, 1H), 3.00–2.87 (m, 1H), 2.78 (s, 2H), 2.56–2.31 (m, 4H), 2.01–1.79 (m, 6H). ^13^C NMR (100 MHz, CDCl_3_) δ 182.8, 164.7, 161.8, 156.7, 156.4, 151.9, 132.2, 130.9, 129.2, 126.4, 108.6, 106.0, 105.3, 101.0, 63.6, 50.3, 49.6, 43.4, 43.0, 26.3, 25.5, 22.7, 22.5. ESI-MS found 449.1, [M + H]^+^.

### 3.2 Prodrug modification improved the solubility of chrysin

The solubility of C-1 in water at pH 7.4 was 3,700 μg/mL at room temperature. the solubility of chrysin in water was 78.4 μg/mL, which is close to the value reported in the literature (0.058 ± 0.04 mg/mL) (Gao et al., 2021). This result suggested that the drawback of the low solubility of chrysin could be improved by adding hydrophilic groups at the C-7 position of chrysin via prodrug modification.

### 3.3 C-1 reduced oxidative stress and cell damage in LO2 cells

**FIGURE 3:**
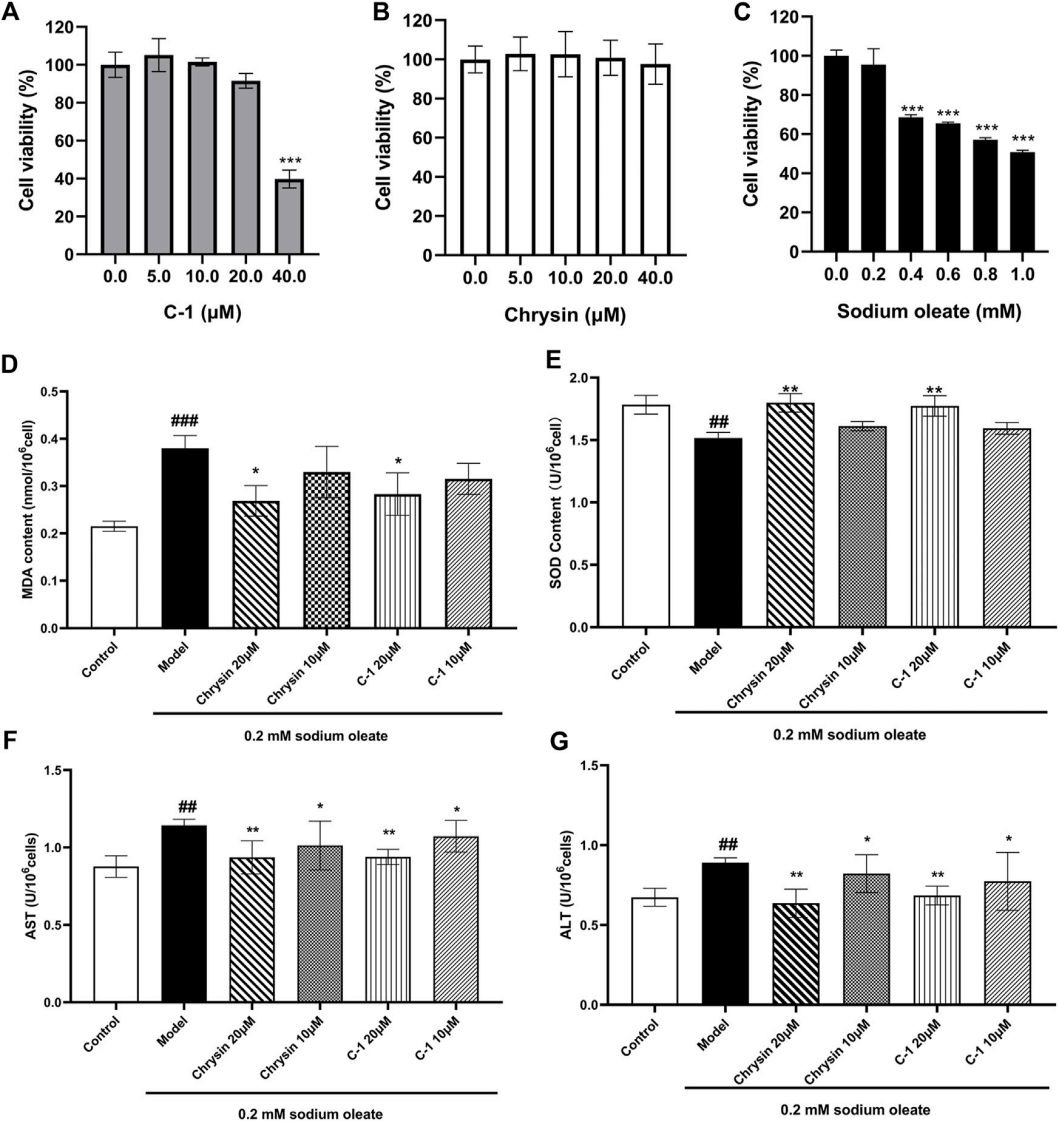
Effects of C-1 on oxidative stress levels and cellular damage in LO2 cells exposed to sodium oleate. **(A-C)** Effect of different concentrations of C-1, chrysin and sodium oleate on cell viability. **(D, E)** Effects of 10 μM and 20 μM of C-1 and chrysin on oxidative stress levels in LO2 cells. **(F, G)** Effects of 10 μM and 20 μM of C-1 and chrysin on cell damage of LO2 cells. ^*^ *p* < 0.05; ^**^ *p* < 0.01, compared with the model group. ^##^ *p* < 0.01; ^###^ *p* < 0.001, compared with the control group.

We examined signature indicators associated with oxidative stress and cellular damage in LO2 cells to explore the protective effect of C-1. LO2 cells exposed to sodium oleate produced significantly increased oxidative stress-related factors compared with those produced by the normal group, and this effect was dose-dependently reversed by C-1 and chrysin treatment (Figures 3D,E). In addition, C-1 and chrysin treatments significantly reduced the abnormally elevated liver enzyme activities of LO2 cells caused by cell injury in a dose-dependent manner (Figures 3F,G). The CCK-8 assay revealed that none of the compounds were obviously toxic to cells at the experimental doses (Figures 3A–C). These results suggest that C-1 reduces oxidative stress and cellular damage in LO2 cells exposed to sodium oleate *in vitro*.

### 3.4 C-1 reduced lipid accumulation in LO2 cells exposed to sodium oleate

**FIGURE 4:**
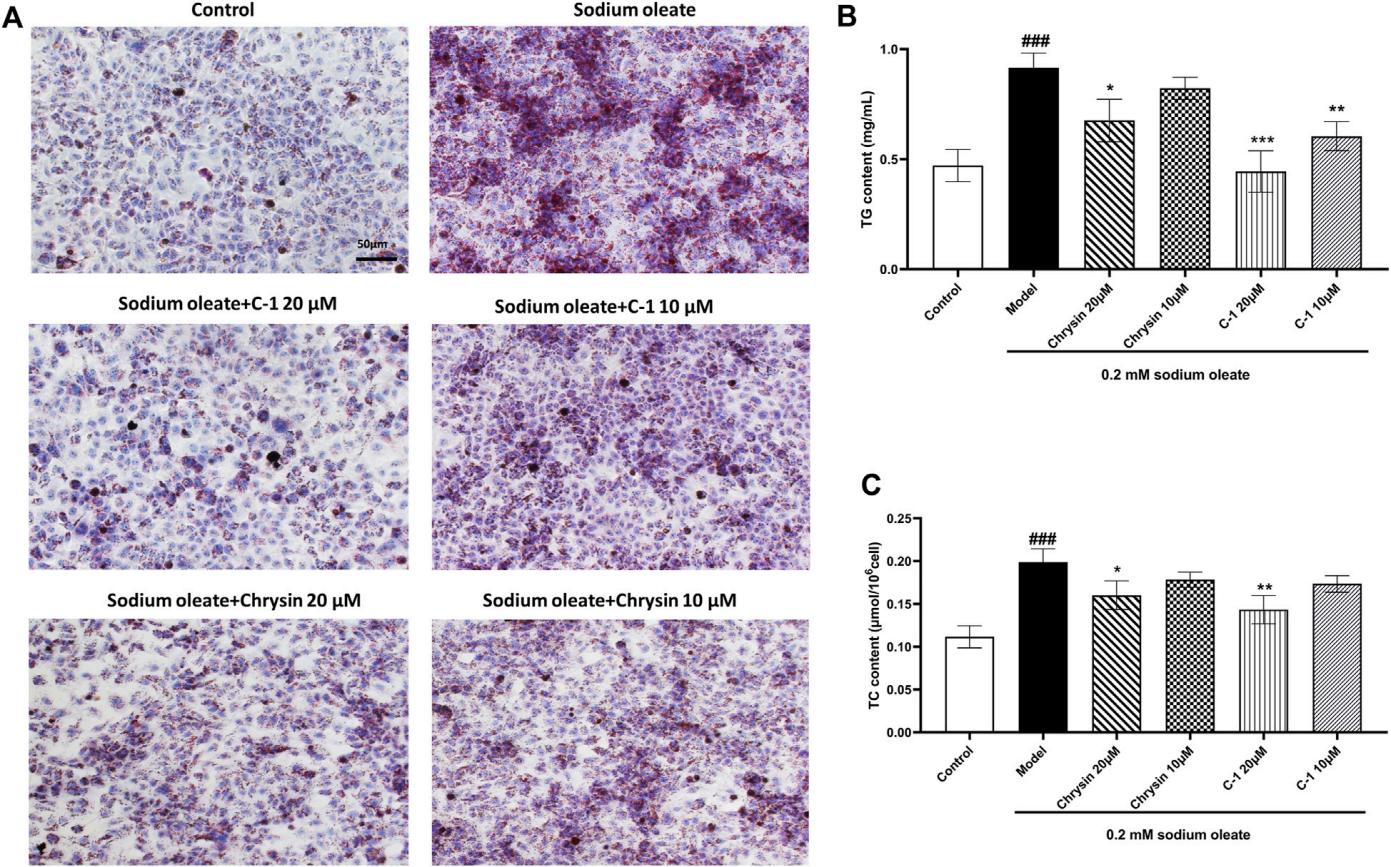
C-1 decreased lipid accumulation in LO2 cells exposed to sodium oleate. **(A)** Oil red O staining showed that C-1 significantly reduced lipid accumulation in LO2 cells exposed to sodium oleate and was more effective than chrysin. Representative images are shown (200×). Scale bar: 50 μm. **(B, C)** C-1 significantly reduced lipid accumulation in LO2 cells exposed to sodium oleate in a dose-dependent manner, as reflected by TG and TC levels. This improvement is superior to the equivalent dose of chrysin. ^*^ *p* < 0.05; ^**^ *p* < 0.01; ^***^ *p* < 0.001, compared with the model group. ^###^ *p* < 0.001, compared with the control group.

As shown in Figure 4, TG, TC, and lipid droplets in LO2 cells were significantly increased when cells were cultured with 0.2 mM sodium oleate, indicating that the NAFLD cell model was successfully constructed. However, this effect was ameliorated in the chrysin and C-1 treatment groups and was more pronounced in the 20 μM C-1 treatment group. These results suggest that C-1 can enhance cellular lipid metabolism *in vitro*.

### 3.5 C-1 reduced the body weight and liver weight of mice

**FIGURE 5:**
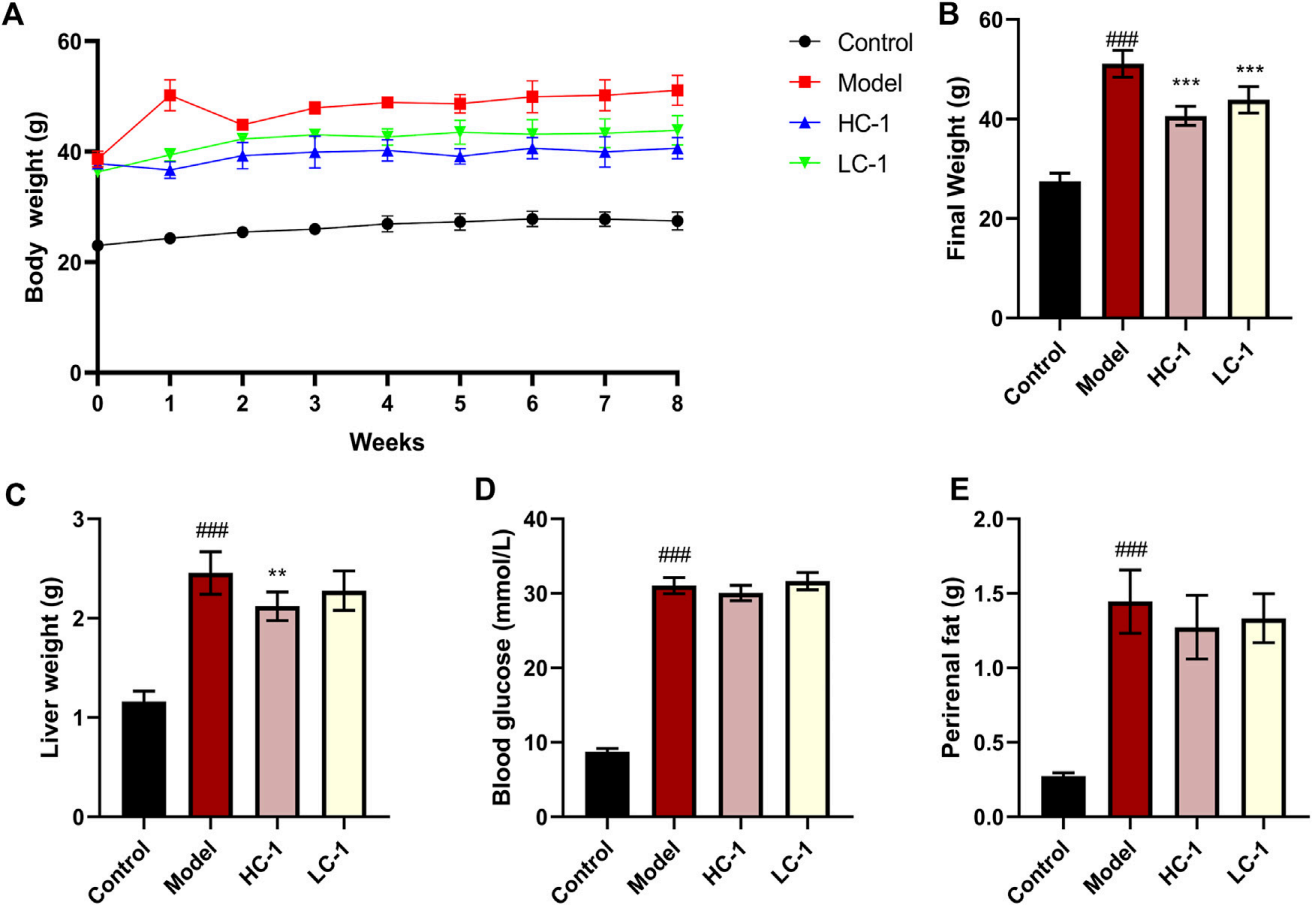
Effects of C-1 treatment on body weight, selected organ weights and blood glucose in mice after 8 weeks of treatment. **(A)** Changes in body weight of mice in each group during 8 weeks; **(B)** body weight of mice in each group at the end of treatment; **(C–E)** Liver weight, blood glucose content and perirenal fat weight of mice in each group at the end of treatment (n = 6). ^**^ *p* < 0.01; ^***^ *p* < 0.001, compared with the model group. ^###^ *p* < 0.001, compared with the control group.

To evaluate the ameliorative effect of C-1 on NAFLD *in vivo*, we selected dbm mice as the control group and db/db mice as the model group. After C-1 feeding for 8 weeks, the rates of body weight gain of db/db mice in the C-1 high-dose treatment group (HC-1, 69.3 mg/kg) and C-1 low-dose treatment group (LC-1, 23.1 mg/kg) were significantly slowed compared with that of the model group (Figure 5A). The final body weight at the end of the treatment was also significantly decreased (Figure 5B). In addition, the liver weight of mice in the HC-1 group was significantly lower than that of mice in the model group (Figure 5C). There were no significant changes in perirenal fat and blood glucose in mice of any tested groups (Figures 5D,E). These results suggest that C-1 may improve NAFLD to a certain extent.

### 3.6 C-1 attenuated liver injury in mice

**FIGURE 6:**
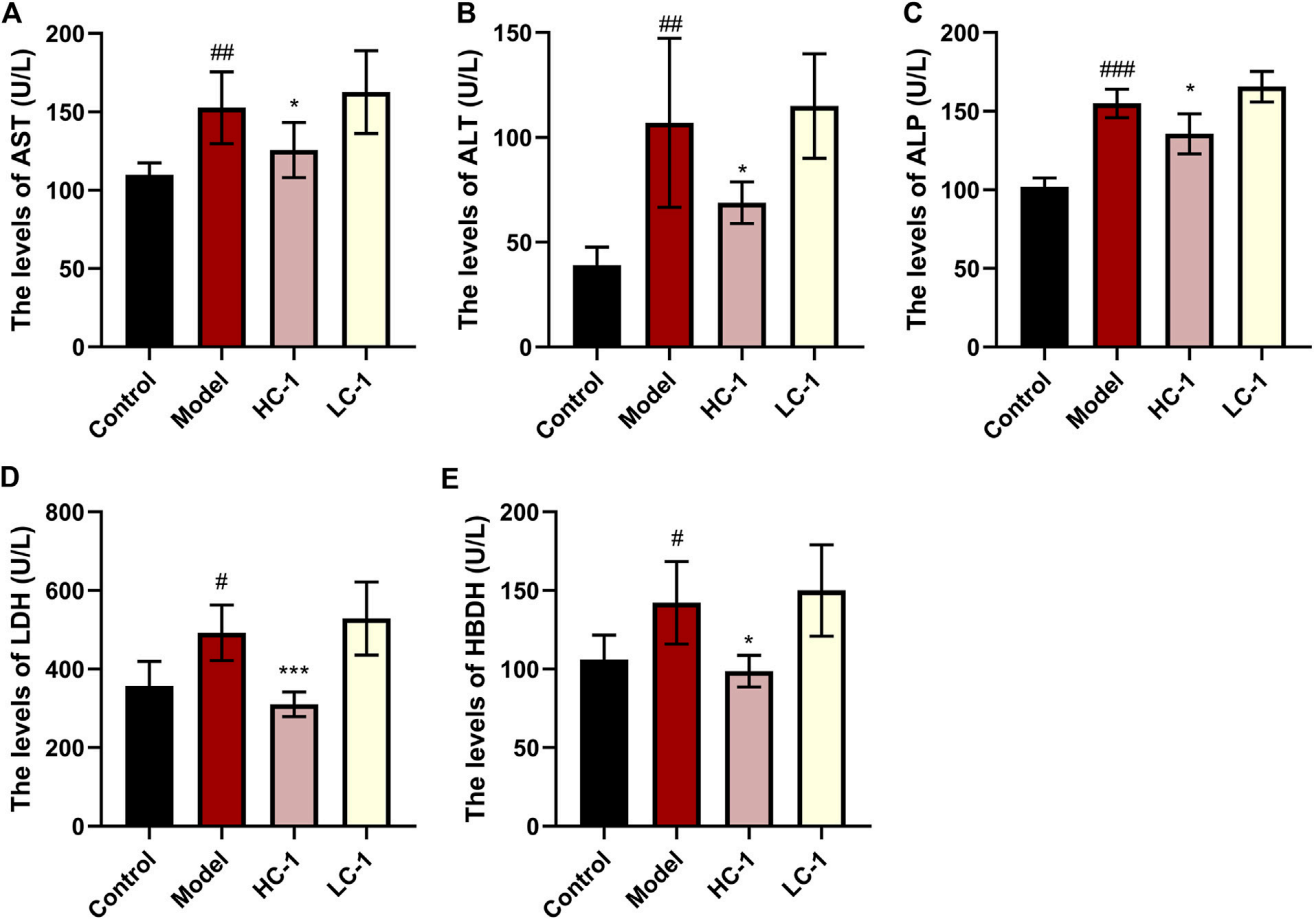
Effect of C-1 on some enzymes related to hepatotoxicity after 8 weeks of treatment (n = 6). ^*^ *p* < 0.05; ^***^ *p* < 0.001, compared with the model group. ^#^ *p* < 0.05; ^##^ *p* < 0.01; ^###^ *p* < 0.001, compared with the control group.

We examined the serum levels of several liver marker enzymes in mice. Elevated activity of these enzymes is thought to be associated with liver injury and disease. As shown in Figure 6, the levels of AST, ALT, ALP, HBDH, and LDH in the model group were significantly elevated compared with those in the control group. The levels of various enzymes were significantly reduced in the HC-1 group, and the LDH and HBDH levels were restored to normal in the HC-1 group after treatment with C-1, but there was no significant change in the LC-1 group. These results indicated that C-1 could alleviate liver injury in NAFLD mice.

### 3.7 C-1 reduced blood lipids in mice

**FIGURE 7:**
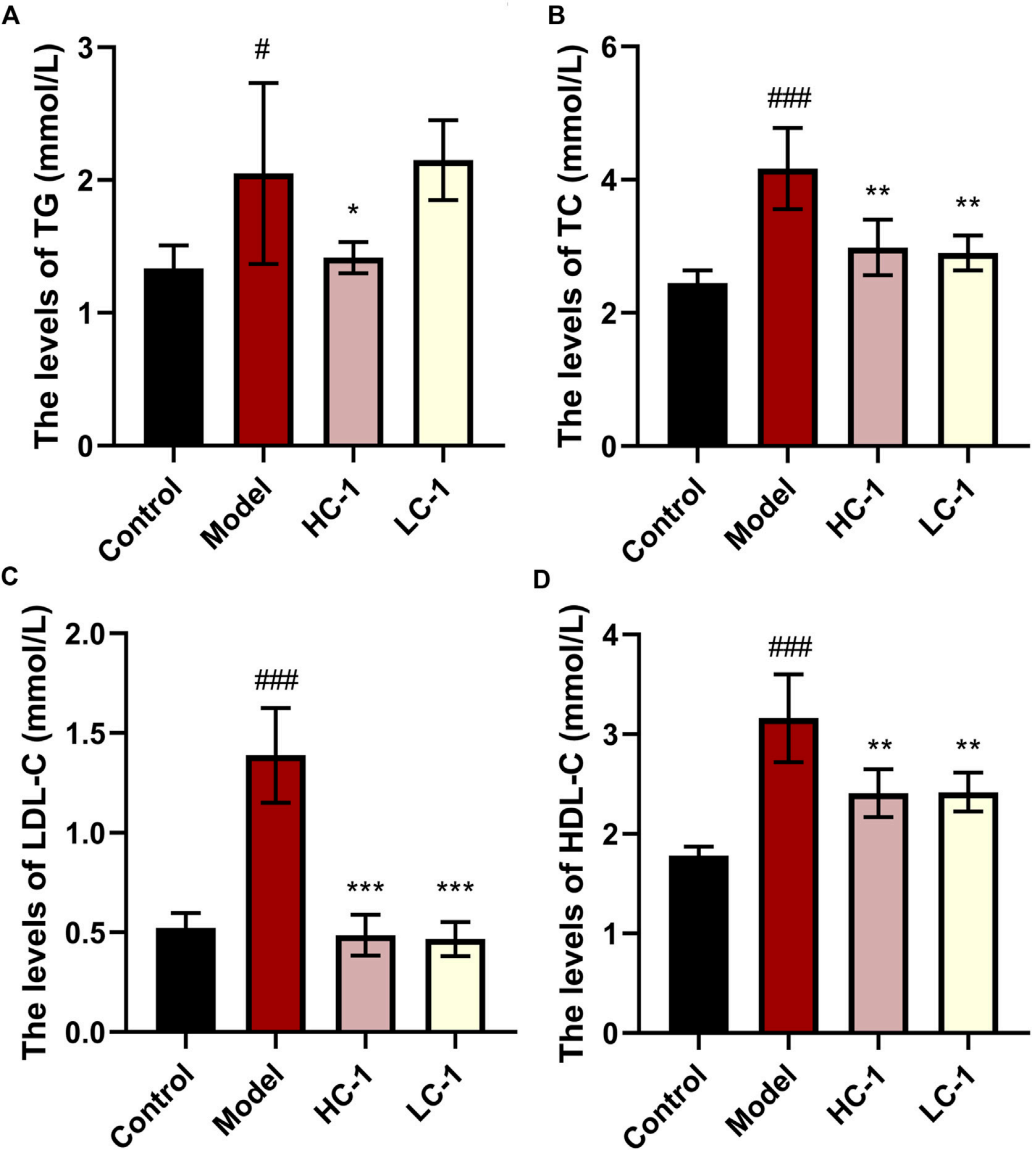
Effect of C-1 on lipid levels of mice after 8 weeks of treatment (n = 6). ^*^ *p* < 0.05; ^**^ *p* < 0.01; ^***^ *p* < 0.001, compared with the model group. ^#^ *p* < 0.05; ^###^ *p* < 0.001, compared with the control group.

As shown in Figure 7, the lipid-related indexes of mice in the model group were significantly elevated compared with those in the control group. After C-1 treatment, the HC-1 group showed significantly reduced TG and TC levels. In addition, TC, low-density lipoprotein cholesterol, and high-density lipoprotein cholesterol levels were significantly reduced in all C-1-administered groups. These results suggest that C-1 can reduce abnormally elevated lipid levels in mice.

### 3.8 C-1 ameliorated hepatic steatosis and enhanced hepatic glycogen storage in db/db mice

**FIGURE 8:**
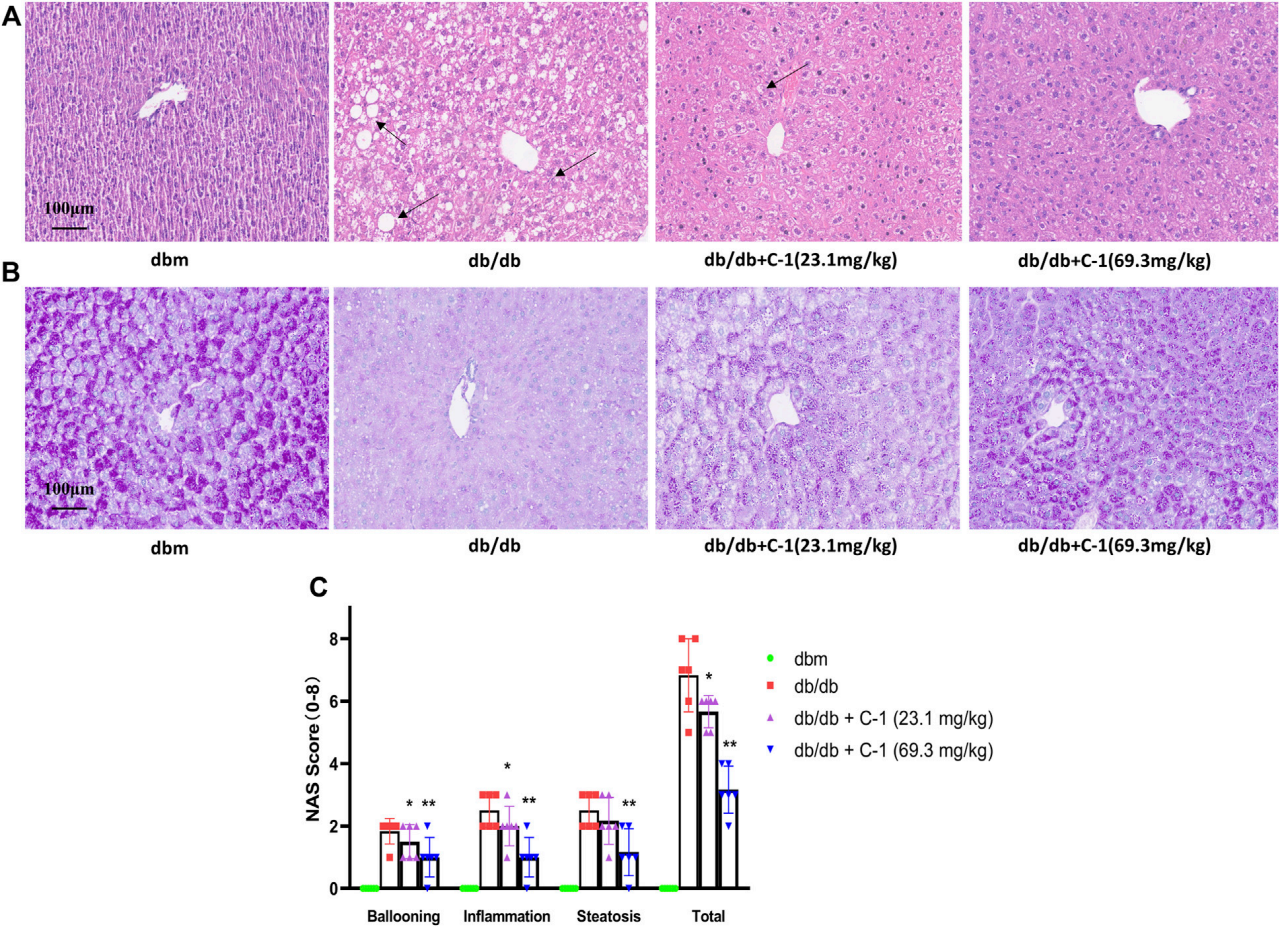
Effects of C-1 on mouse liver histology after 8 weeks of treatment (n = 6). Representative images were shown (20 ×). Scale bar: 100 μm. **(A)** HE staining results of liver tissue; **(B)** PAS staining results of liver tissue; **(C)** A semi-quantitative approach was used to grade histological sections under the microscope. ^*^ *p* < 0.05; ^**^ *p* < 0.01.

HE staining of liver histological sections of db/db mice showed significant steatosis, severe mononuclear macrophage infiltration, and apoptosis of liver cells. These abnormalities were reduced by C-1 treatment (Figure 8A). In addition, periodic acid-Schiff staining showed a significant reduction in the glycogen content of purple-stained liver cells in db/db mice, which was reversed by C-1 treatment; this improvement was more pronounced in the high-dose (69.3 mg/kg) condition (Figure 8B). Grading of the histological sections indicated C-1 treatment significantly reduced the NAFLD activity score (NAS) in a dose-dependent manner (Figure 8C). These results suggested that C-1 ameliorates hepatic steatosis and hepatocyte apoptosis in mice caused by NAFLD and reverses the reduction of hepatic glycogen.

### 3.9 C-1 treatment modified abnormal proteomic profiles in the liver of db/db mice

**FIGURE 9:**
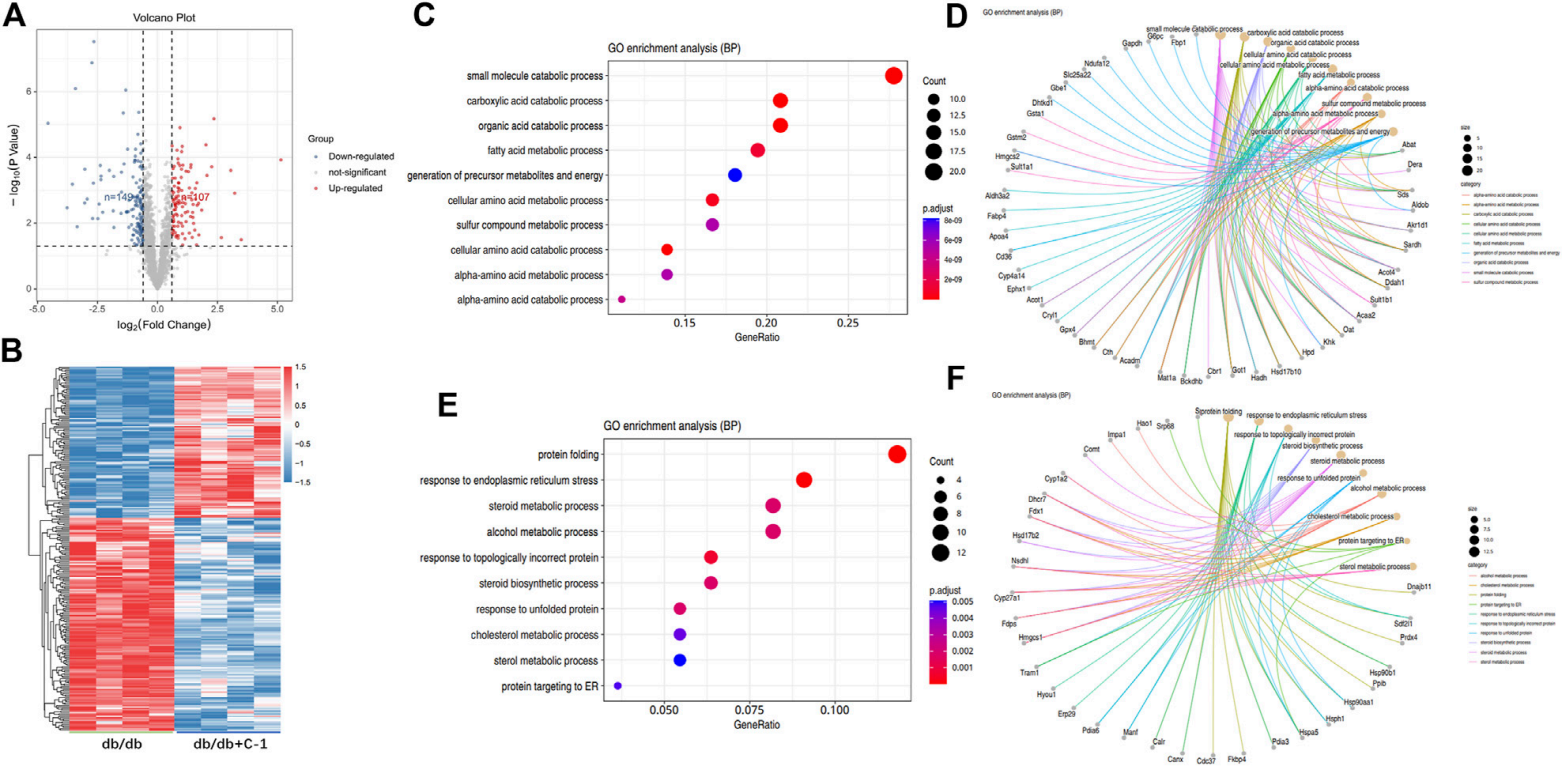
GO analysis of differentially expressed proteins in db/db + C-1 vs. db/db mice. **(A)** DE proteins analyzed by the volcano plot and **(B)** the heatmap. The color of blue represented low abundance, red represented high abundance (n = 4 mice/group). **(C, D)** GO enrichment BP analysis for upregulated DEPs. **(E, F)** GO enrichment BP analysis for downregulated DEPs.

The TMT labeling technique and LC-MS/MS analysis were combined to capture 1931 proteins in the liver. To explore the molecular mechanism of C-1 in improving the liver function of db/db mice, we further analyzed the differentially expressed proteins (DEPs) in the liver between untreated db/db mice and db/db mice with C-1 treatment. Compared with untreated db/db mice, 256 DEPs were detected in db/db mice with C-1 treatment, of which 149 DEPs were upregulated and 107 were downregulated (Figures 9A,B). The GO enrichment analysis results showed that these DEPs were mainly associated with metabolic processes, catabolic processes, cellular processes, biosynthesis, and protein reactions. The upregulated DEPs were mainly involved in small molecule catabolic processes, carboxylic acid catabolic processes, organic acid catabolic processes, and fatty acid metabolic processes (Figures 9C,D). The downregulated DEPs were mainly involved in protein folding, response to endoplasmic reticulum stress, steroid metabolic processes, and alcohol metabolic processes (Figures 9E,F). The results suggest that C-1 treatment may activate several metabolic processes in the liver of db/db mice and positively affect obesity and fat accumulation caused by metabolic dysfunction.

**FIGURE 10:**
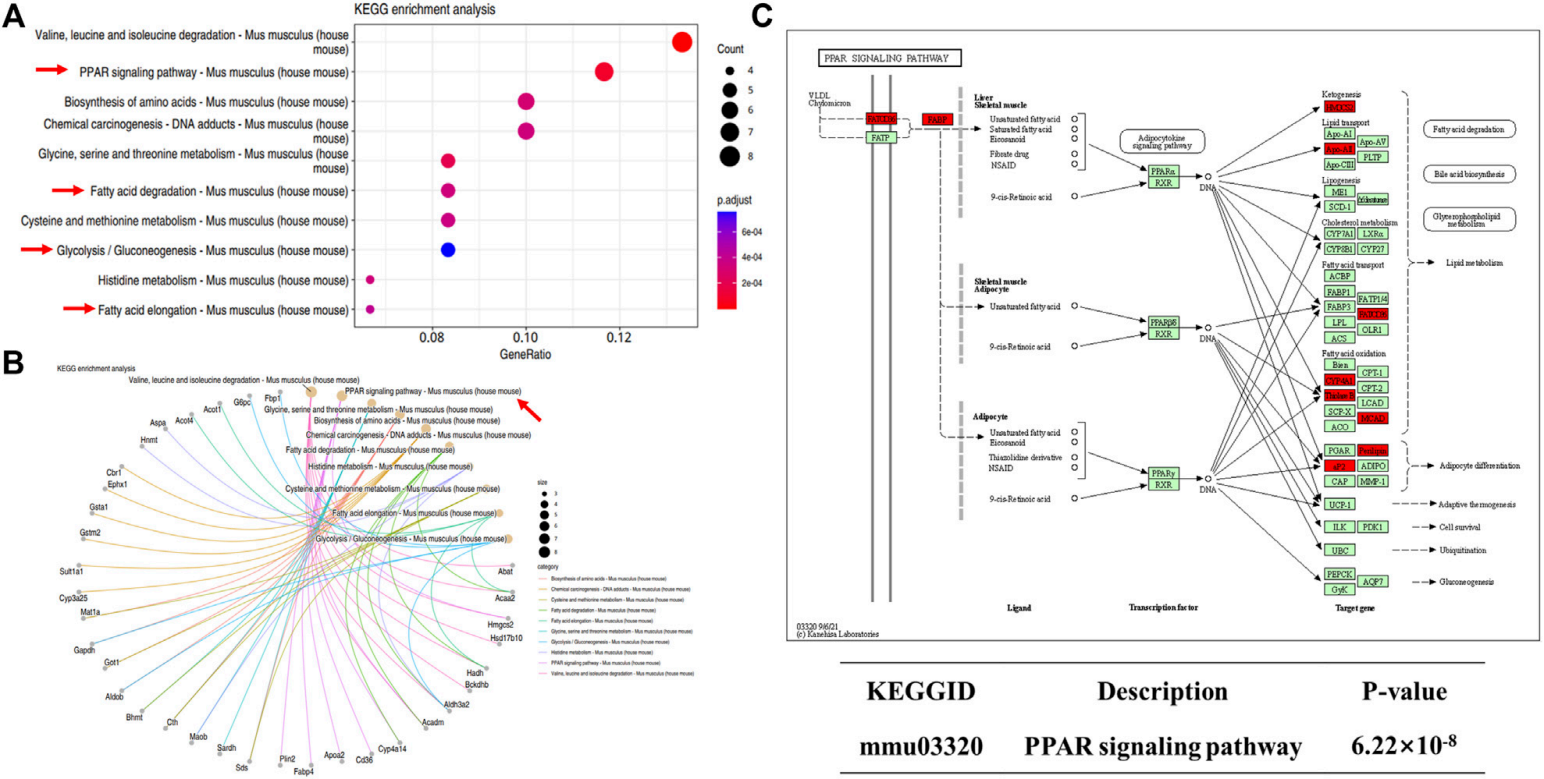
KEGG analysis of differentially expressed proteins in db/db + C-1 vs. db/db mice. **(A, B)** KEGG analysis for upregulated DEPs. **(C)** PPAR signaling pathway and its upregulated proteins (marked in red).

We next performed KEGG enrichment analysis of DEPs. We found 10 major signaling pathways involving upregulated DEPs, among which those directly related to NAFLD included the peroxisome proliferator-activated receptor (PPAR) signaling pathway, glycolysis/gluconeogenesis, fatty acid degradation, and fatty acid elongation (Figures 10A,B). The most enriched DEPs were involved in the PPAR signaling pathway (Figure 10C), which includes key proteins such as scavenger receptor B2 (CD36), fatty acid binding protein 4 (Fabp4), and the apolipoprotein A-II (Apoa2). PPARs are thought to play a key role in the pathology of NAFLD, and PPAR agonists are considered promising agents for the treatment of NAFLD (Boeckmans et al., 2019; Gawrieh et al., 2021). PPARs are ligand-activated transcription factors of the nuclear receptor superfamily and are involved in the transcriptional regulation of lipid metabolism, glucose homeostasis, energy homeostasis, inflammation, and atherosclerosis (Wagner and Wagner, 2020). Recent studies have shown that the PPAR signaling pathway also plays an important role in metabolic disorders such as obesity (Faghfouri et al., 2021) and type 2 diabetes (Ji et al., 2022). The above proteomics results suggest that C-1 may improve liver function in db/db mice by altering the abnormal protein network and activating signaling pathways such as PPAR.

### 3.10 C-1 improved the pharmacokinetic properties of chrysin

**FIGURE 11:**
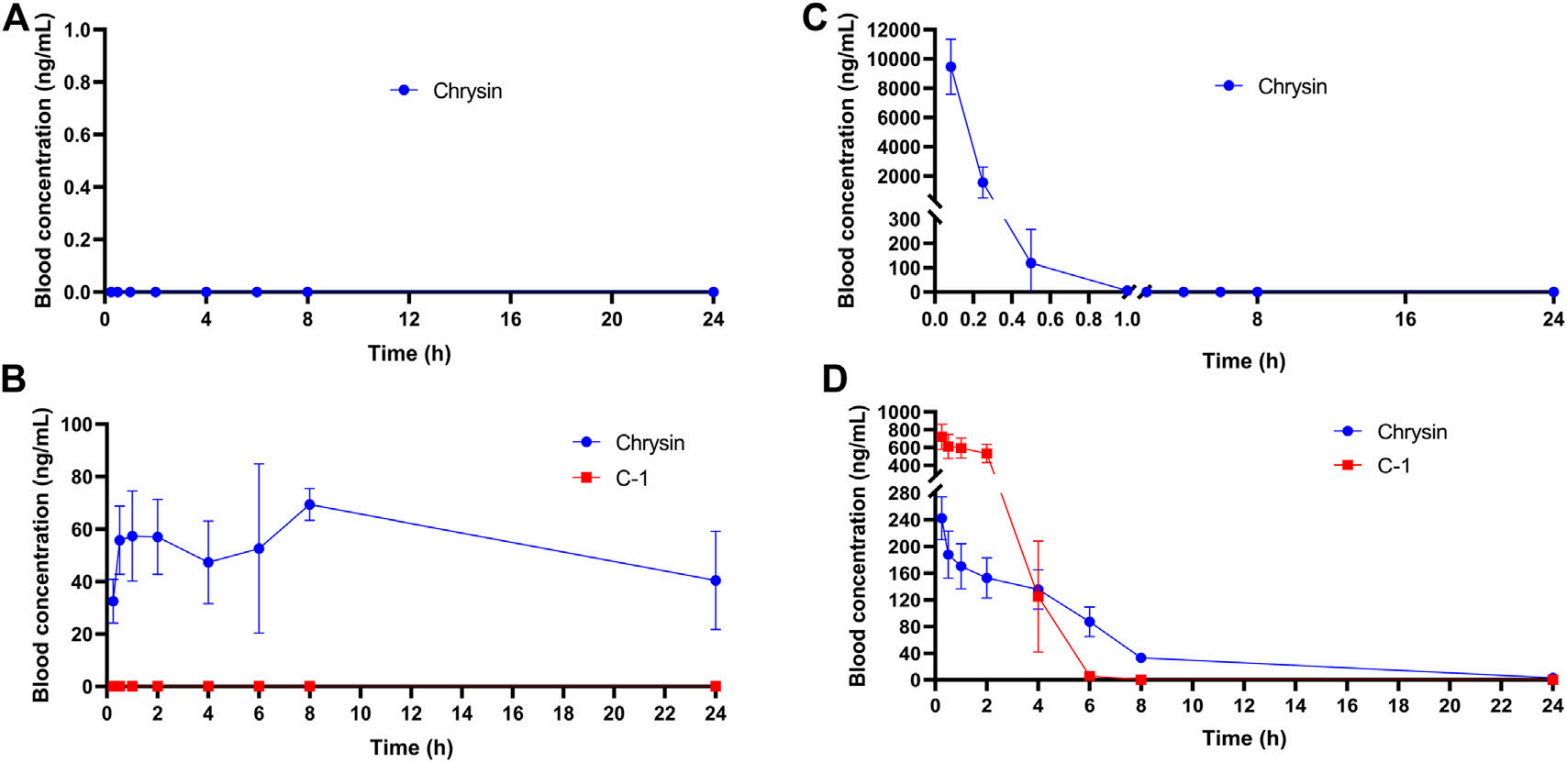
Plasma concentration-time curves of **(A)** Chrysin (50.0 mg/kg) after a p.o. administration in rats; **(B)** C-1 (95.4 mg/kg) after a p.o. administration in rats; **(C)** Chrysin (10 mg/kg) after an i.v. administration in rats; **(D)** C-1 (19.1 mg/kg) after an i.v. administration in rats (n = 3). The pharmacokinetic parameters were calculated by Phoenix WinNonlin7.0.

The plasma stability has a significant influence on the concentration of drug available in the circulation. We assessed the concentration and initial metabolism of chrysin and C-1 administered by gavage or intravenously in plasma. After administration of chrysin by gavage, it was undetectable in plasma samples collected at all blood collection points (Figure 11A). In contrast, after gavage administration of C-1, chrysin (>20 ng/mL) was detectable in plasma samples collected at the first blood collection point (0.083 h) and remained at a certain concentration (20–80 ng/mL) until the endpoint of the experiment (24 h) (Figure 11B). Notably, C-1 was never detectable in plasma during the process. This finding suggests that C-1, as a prodrug, can be rapidly metabolized to its original molecule, chrysin, under gavage administration conditions. Intravenously administered chrysin was not detectable in plasma samples collected at the blood collection site after 1 h (Figure 11C). In contrast, intravenous administration of C-1 enabled chrysin to remain detectable in plasma samples collected at 8 h (Figure 11D). Analysis of pharmacokinetic parameters showed that C-1, as a prodrug, prolonged the half-life (t_1/2_) of its original molecule, chrysin, to 16.73 ± 8.37 h under gavage administration and to 4.12 ± 0.59 h under i.v. administration (t_1/2_ for chrysin 0 h and 0.17 ± 0.14 h, respectively). In addition, C-1 increased the oral bioavailability (F) of chrysin to 24.22% ± 2.59%. Other pharmacokinetic parameters are provided in the Supplementary Table S1–S6. These results suggest that C-1 is effective as a prodrug to improve the pharmacokinetic properties of its original molecule, chrysin.

### 3.11 C-1 had a low toxicity profile

In the acute toxicity test, rats did not show any abnormal behavior, and no deaths were recorded after a single p.o. administration of 500 mg/kg or 1,000 mg/kg of C-1. The rats exhibited red nasal secretions and a significant decrease in body weight and food intake after p.o. administration of 2000 mg/kg of C-1. These data suggest that the maximum tolerated dose of C-1 in rats is 1,000 mg/kg (n = 3). Specific details are provided in the (Supplementary Table S7, S8).

## 4 Discussion

Currently, there are still no approved drugs for the treatment of NAFLD. Notably, resmetirom, a thyroid hormone receptor-β agonist developed by Madrigal for the treatment of NASH with liver fibrosis, was granted breakthrough therapy designation in April 2023 by the FDA (Karim and Bansal, 2023; Wang et al., 2023). Otherwise, the current state of drug development for NAFLD remains unsatisfactory (Banini and Sanyal, 2017; Fang et al., 2022; Umemura et al., 2022). Chrysin has been reported to have potential for NAFLD treatment; however, the low bioavailability of chrysin limits its efficacy. Therefore, its chemical modification may be a viable strategy to improve its drug-forming properties. From a chemical point of view, the 5-O and 7-O positions of chrysin are key points for its easy modification, and the oxygen atom-free B and C rings allow it to act as a hydrophobic center in many biological interactions (Falbo and Aiello, 2023). In recent years, researchers have developed several chrysin derivatives with modification of the 7-O position that exhibit anti-inflammatory (Cho et al., 2004), antioxidant (Zhu et al., 2016), antitumor (Liu et al., 2020), and antidiabetic (Zhang et al., 2021) activity. Conformational studies of existing novel chrysin derivatives have shown that modification of the 7-O site of chrysin can enhance its biological activity and improve its pharmacokinetic properties (Müller-Schiffmann et al., 2010; Singh et al., 2015), which aroused our interest.

In the present study, we focused on improving the oral bioavailability and bioactivity of chrysin. We synthesized a novel chrysin derivative, C-1, by introducing a bipiperidine structure into the 7-O position of chrysin and hydrochlorinating it. Interestingly, the introduction of this structure increased the solubility of the compound in water by approximately 47 times compared with that of chrysin. We next focused on the ameliorative effects of C-1 on sodium oleate-induced LO2 cells *in vitro*. C-1 significantly ameliorated damage, lipid accumulation, and oxidative stress levels in LO2 cells, suggesting that it has relatively substantial *in vitro* activity as a prodrug, which is somewhat unusual (prodrugs are generally considered to have low activity or be inactive *in vitro*). We hypothesize that, on the one hand, the carbamate structure of C-1 can be rapidly metabolized and partially release chrysin via enzymes in hepatocytes. On the other hand, the simple chemical modification of the 7-O position did not affect the groups in the structure of chrysin that bear relevant biological functions (e.g., the carbonyl group on C-4, the carbon-carbon double bond between C-2 and C-3, the phenolic hydroxyl group at position 5, and the unaffected B and C rings).

According to earlier published studies, one of the reasons for the low oral bioavailability of chrysin may be the rapid elimination of its bound metabolites caused by efflux transporter proteins in the liver and intestines, such as MRP2 and BCRP (Walle et al., 1999; Ge et al., 2015), as these metabolic conjugates are water soluble. Our rat pharmacokinetic experimental data for C-1 and chrysin indicated that C-1 was metabolized to chrysin so rapidly upon oral administration that C-1 was undetectable at all blood collection sites. This ability of C-1 to be rapidly metabolized into active molecules may be why it remains somewhat active *in vitro*. In addition, our chemical modification of the 7-O position significantly increased the oral bioavailability (F = 24.22 ± 2.59%) and t_1/2_ (16.73 ± 8.37 h) of chrysin. In a previous study, we found that the continuous addition of chrysin at a dose of 40 mg/kg/d to a high-fat diet in mice administered for 21 weeks significantly reduced final body weight and liver weight and improved hepatic lipid metabolism compared to mice provided with only a high-fat diet (Gao et al., 2023). Remarkably, in the present study, C-1, as a prodrug of chrysin, achieved close therapeutic efficacy at a shorter treatment period (8 weeks) and lower administered dose (69.3 mg/kg/d, which is equivalent to chrysin administered at a dose of 36.3 mg/kg/d when converted on a molar ratio basis). Notably, this prodrug appears to be a slow-release vehicle that provides a sustained supply of precursors, as evidenced by chrysin maintaining a high blood concentration (>40 ng/mL) for 24 h. The pharmacokinetic results provided a rationale for the *in vivo* anti-NAFLD activity of C-1. We found that a high dose (69.3 mg/kg) of C-1 significantly reduced the abnormally elevated body weights of db/db mice and significantly reduced their liver weights, although these indices did not return to the levels of those in the normal group. In addition, C-1 had a significant lowering effect on TG and TC levels in mice, and this effect was particularly pronounced for TC, suggesting an anti-hyperlipidemic effect of C-1. Notably, C-1 concurrently reduced HDL-C levels in db/db mice because of over compensatory behavior, and this type of cholesterol is generally considered to be beneficial. C-1 also reduced serum levels of AST, ALT, ALP, LDH, and HBDH. Elevated levels of these enzymes indicate liver damage, and C-1 reversed this change. Notably, these therapeutic effects were dose-related. The NAS is the “gold standard” for NAFLD diagnosis. The NAS and HE staining results visually verified the ameliorative effect of C-1 on NAFLD in mice. Interestingly, periodic acid-Schiff staining showed that C-1 increased hepatic glycogen content. Decreased hepatic glycogen content is an important feature of insulin resistance, which leads to the transfer of unstorable glucose to fat synthesis and exacerbates steatosis in NAFLD (Irimia et al., 2017). The above results suggested that C-1 may ameliorate NAFLD in mice through its anti-hyperlipidemia efficacy and improvement of insulin resistance.

Hepatic lipid metabolism plays a key role in the development of NAFLD. Proteomic analysis showed that C-1 treatment enhanced processes such as fatty acid, amino acid, and cellular metabolism, which contributed to our understanding of the mechanism by which C-1 improved hepatic lipid metabolism in db/db mice. Our analysis showed that C-1 activated nine key proteins in the PPAR signaling pathway, suggesting that C-1 may regulate hepatic lipid metabolism by activating PPAR signaling in the liver of db/db mice. In addition, some of the proteins involved in other related metabolic pathways (glucose metabolism and fatty acid catabolism) were also increased by C-1 treatment. These results reveal that C-1 may ameliorate hepatic lipid accumulation by strengthening the metabolic cycle in the liver.

In summary, we utilized the carbamate structure to protect the 7-O position of chrysin and hydrochloride it, thereby improving the solubility and bioavailability of chrysin. Additional studies revealed that C-1 demonstrated favorable anti-NAFLD activity in ameliorating lipid accumulation, cellular damage, and oxidative stress in LO2 cells *in vitro*. In addition, high-dose C-1 (69.3 mg/kg) significantly reduced body weight and liver weight and ameliorated liver injury and hyperlipidemia in db/db mice *in vivo*. Liver histopathology showed that C-1 significantly reduced the NAS and improved insulin resistance in mice. Acute toxicity experiments also showed the safety of C-1 at higher doses (1,000 mg/kg). Proteomic analysis indicated that C-1 may improve hepatic lipid metabolism in mice by activating hepatic signaling pathways associated with the lipid metabolic cycle. Pharmacokinetic studies showed that C-1 was rapidly metabolized *in vivo* to its original molecule, chrysin, and C-1 significantly improved the metabolic properties and bioavailability of chrysin. This study provides an experimental basis and new ideas for developing safer drugs for clinical application for treatment of NAFLD.

## Data Availability

The datasets presented in this study can be found in online repositories and Supplementary Material. The data have been deposited to the ProteomeXchange Consortium (http://proteomecentral.proteomexchange.org) via the iProX partner repository with the accession number PXD051284.
