# Supplementary material for: Synthesis and biological evaluation of the novel chrysin prodrug for non-alcoholic fatty liver disease treatment
Source: Front Pharmacol. 2024 Apr 19;15:1336232. doi: 10.3389/fphar.2024.1336232 (PMC11066169; doi:10.3389/fphar.2024.1336232)
Supplement: Supplementary file 2 [file DataSheet2.docx]

***Supplementary Material***


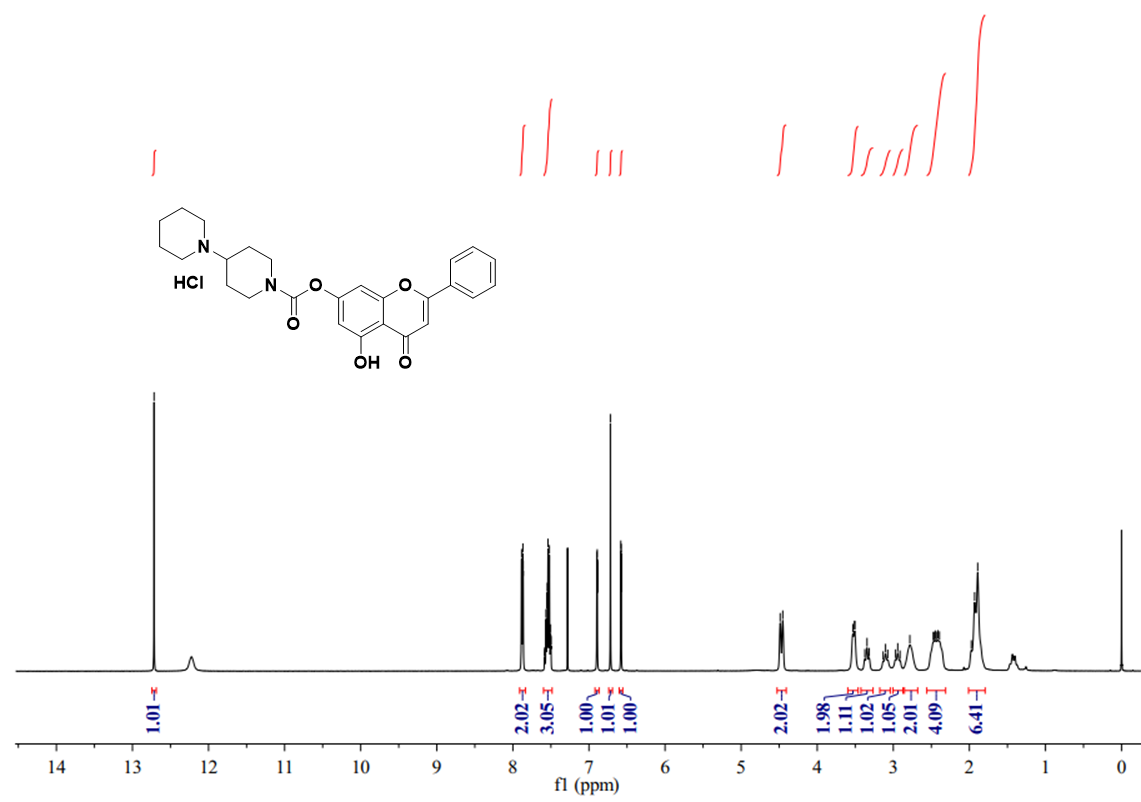


**Supplementary Figure 1.** ^1^H NMR of C-1.


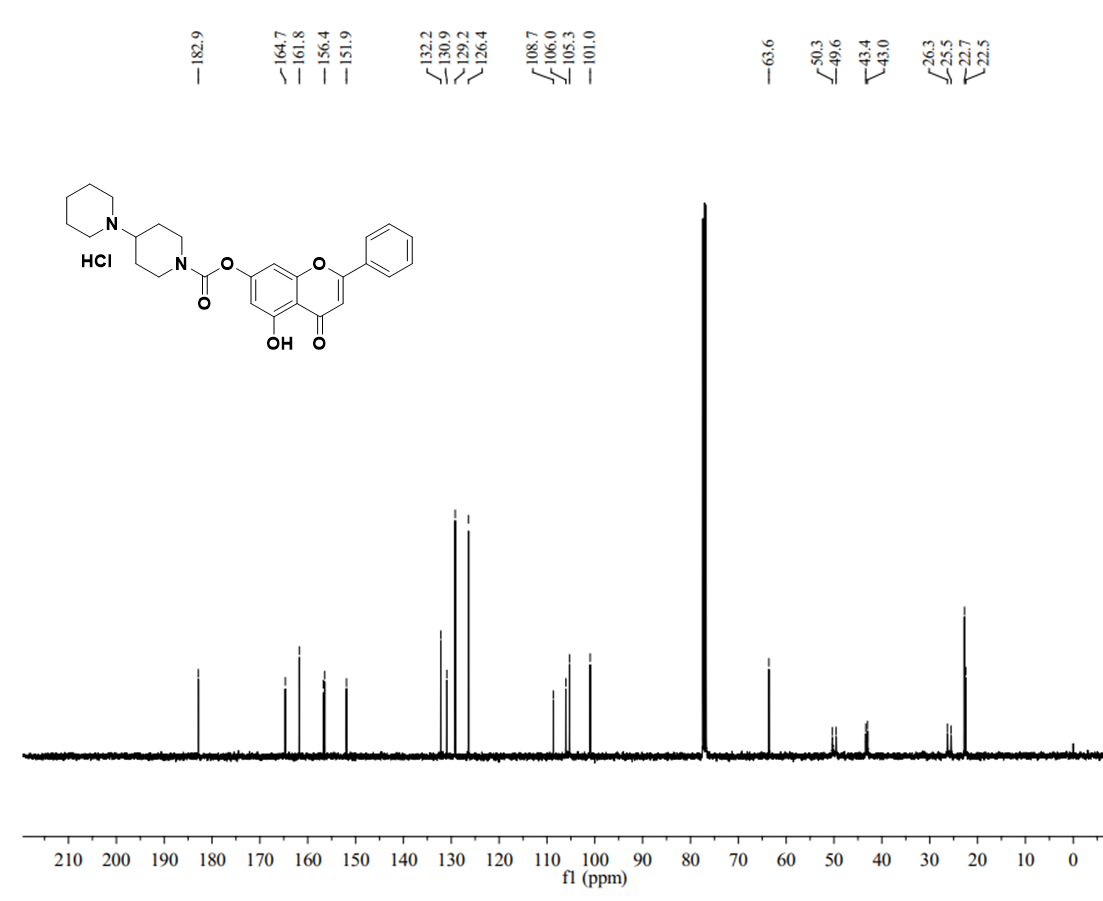


**Supplementary Figure 2.** ^13^C NMR of C-1.


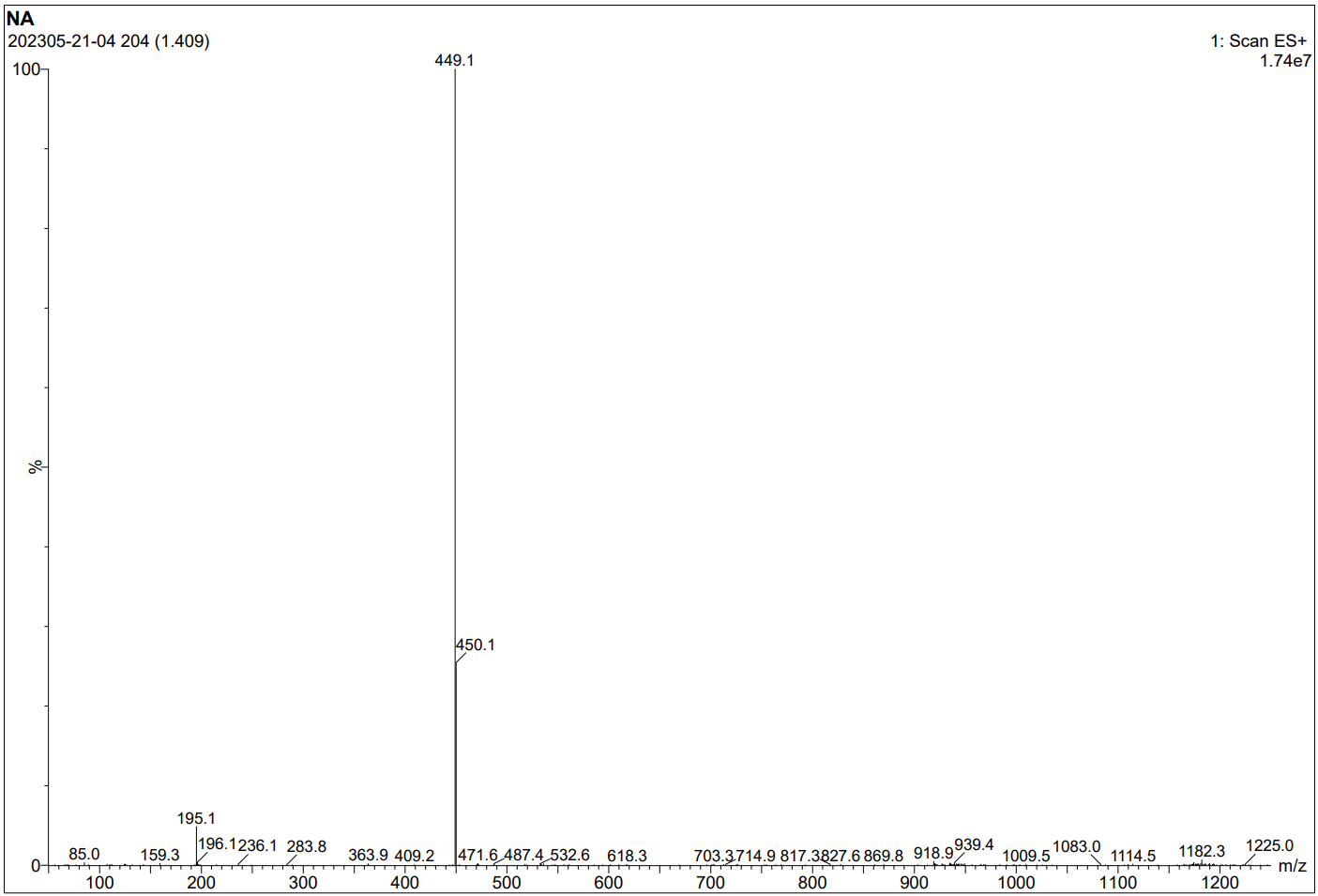


**Supplementary Figure 3.** MS of C-1.

**Supplementary Table 1.** PK parameters of chrysin administered by p.o.

| Compound | Parameters | Unit | 201 | 202 | 203 | Mean ± SD |
| --- | --- | --- | --- | --- | --- | --- |
| **Chrysin** | Dose | mg/kg | 50.00 | 50.00 | 50.00 | 50.00 ± 0 |
|  | AUC_(0-t)_ | ng/mL*h | 9.61 | NA | NA | NA |
|  | AUC_(0-∞)_ | ng/mL*h | NA | NA | NA | NA |
|  | MRT_(0-t)_ | h | 8.00 | NA | NA | NA |
|  | MRT_(0-∞)_ | h | NA | NA | NA | NA |
|  | T_1/2_ | h | NA | NA | NA | NA |
|  | T_max_ | h | 8.00 | NA | NA | NA |
|  | C_max_ | ng/mL | 2.04 | NA | NA | NA |
|  | F | % | 0.07 | NA | NA | NA |

**Supplementary Table 2.** PK parameters of C-1 administered by p.o. (detection of chrysin).

| Compound | Parameters | Unit | 201 | 202 | 203 | Mean ± SD |
| --- | --- | --- | --- | --- | --- | --- |
| **Chrysin** | Dose | mg/kg | 95.40 | 95.40 | 95.40 | 95.40 ± 0 |
|  | AUC_(0-t)_ | ng/mL*h | 1223.70 | 1226.66 | 1466.53 | 1305.63 ± 139.35 |
|  | AUC_(0-∞)_ | ng/mL*h | 1643.89 | 2290.01 | NA | 1966.95 ± 456.88 |
|  | MRT_(0-t)_ | h | 9.59 | 9.79 | 12.46 | 10.61 ± 1.60 |
|  | MRT_(0-∞)_ | h | 17.26 | 31.56 | NA | 24.41 ± 10.11 |
|  | T_1/2_ | h | 10.81 | 22.65 | NA | 16.73 ± 8.37 |
|  | T_max_ | h | 6.00 | 1.00 | 8.00 | 5.00 ± 3.61 |
|  | C_max_ | ng/mL | 89.81 | 76.65 | 75.07 | 80.51 ± 8.09 |
|  | F | % | 22.70 | 22.76 | 27.21 | 24.22 ± 2.59 |

**Supplementary Table 3.** PK parameters of C-1 administered by p.o.

| Compound | Parameters | Unit | 201 | 202 | 203 | Mean ± SD |
| --- | --- | --- | --- | --- | --- | --- |
| **C-1** | Dose | mg/kg | 95.40 | 95.40 | 95.40 | 95.40 ± 0 |
|  | AUC_(0-t)_ | ng/mL*h | NA | 0.79 | 96.16 | 48.47 ± 67.44 |
|  | AUC_(0-∞)_ | ng/mL*h | NA | NA | NA | NA |
|  | MRT_(0-t)_ | h | NA | 0.50 | 8.00 | 4.25 ± 5.30 |
|  | MRT_(0-∞)_ | h | NA | NA | NA | NA |
|  | T_1/2_ | h | NA | NA | NA | NA |
|  | T_max_ | h | NA | 0.50 | 8.00 | 4.25 ± 5.30 |
|  | C_max_ | ng/mL | NA | 3.14 | 24.04 | 13.59 ± 14.78 |
|  | F | % | NA | 0.01 | 1.83 | 0.92 ± 1.28 |

**Supplementary Table 4.** PK parameters of chrysin administered by i.v.

| Compound | Parameters | Unit | 101 | 102 | 103 | Mean ± SD |
| --- | --- | --- | --- | --- | --- | --- |
| **Chrysin** | Dose | mg/kg | 10.00 | 10.00 | 10.00 | 10.00 ± 0 |
|  | AUC_(0-t)_ | ng/mL*h | 2095.00 | 2971.49 | 2693.88 | 2586.79 ± 447.95 |
|  | AUC_(0-∞)_ | ng/mL*h | 2095.21 | 2972.15 | 2694.45 | 2587.27 ± 448.19 |
|  | MRT_(0-t)_ | h | 0.06 | 0.11 | 0.07 | 0.08 ± 0.03 |
|  | MRT_(0-∞)_ | h | 0.06 | 0.11 | 0.07 | 0.08 ± 0.03 |
|  | T_1/2_ | h | 0.09 | 0.33 | 0.08 | 0.17 ± 0.14 |
|  | T_max_ | h | 0.08 | 0.08 | 0.08 | 0.08 ± 0 |
|  | Vss | mL/kg | 268.42 | 374.21 | 249.39 | 297.34 ± 67.25 |
|  | Vz | mL/kg | 615.06 | 1579.60 | 448.97 | 881.21 ± 610.50 |
|  | CL | mL/h/kg | 4772.80 | 3364.57 | 3711.33 | 3949.57 ± 733.72 |
|  | C_max_ | ng/mL | 7344.09 | 10992.90 | 10049.40 | 9462.13 ± 1893.97 |

**Supplementary Table 5.** PK parameters of C-1 administered by i.v. (detection of chrysin).

| Compound | Parameters | Unit | 101 | 102 | 103 | Mean ± SD |
| --- | --- | --- | --- | --- | --- | --- |
| **Chrysin** | Dose | mg/kg | 19.08 | 19.08 | 19.08 | 19.08 ± 0 |
|  | AUC_(0-t)_ | ng/mL*h | 1217.11 | 1089.11 | 927.83 | 1078.02 ± 144.96 |
|  | AUC_(0-∞)_ | ng/mL*h | 1227.92 | 1117.23 | 941.36 | 1095.51 ± 144.51 |
|  | MRT_(0-t)_ | h | 4.08 | 5.14 | 4.16 | 4.46 ± 0.59 |
|  | MRT_(0-∞)_ | h | 4.30 | 5.78 | 4.53 | 4.87 ± 0.80 |
|  | T_1/2_ | h | 3.49 | 4.65 | 4.22 | 4.12 ± 0.59 |
|  | T_max_ | h | 0.083 | 0.083 | 0.083 | 0.08 ± 0 |
|  | Vss | mL/kg | 66765.29 | 98740.43 | 91910.55 | 85805.43 ± 16839.15 |
|  | Vz | mL/kg | 78248.19 | 114583.91 | 123361.30 | 105397.80 ± 23918.33 |
|  | CL | mL/h/kg | 15538.47 | 17077.91 | 20268.47 | 17628.28 ± 2412.55 |
|  | C_max_ | ng/mL | 244.23 | 209.56 | 273.07 | 242.28 ± 31.80 |

**Supplementary Table 6.** PK parameters of C-1 administered by i.v.

| Compound | Parameters | Unit | 101 | 102 | 103 | Mean ± SD |
| --- | --- | --- | --- | --- | --- | --- |
| **C-1** | Dose | mg/kg | 19.08 | 19.08 | 19.08 | 19.08 ± 0 |
|  | AUC_(0-t)_ | ng/mL*h | 1135.70 | 1311.16 | 707.28 | 1051.38 ± 310.65 |
|  | AUC_(0-∞)_ | ng/mL*h | 1139.83 | 1315.89 | 744.68 | 1066.80 ± 292.52 |
|  | MRT_(0-t)_ | h | 0.89 | 0.99 | 0.68 | 0.86 ± 0.16 |
|  | MRT_(0-∞)_ | h | 0.92 | 1.01 | 0.78 | 0.90 ± 0.11 |
|  | T_1/2_ | h | 0.88 | 0.46 | 0.46 | 0.60 ± 0.24 |
|  | T_max_ | h | 0.083 | 0.083 | 0.083 | 0.08 ± 0 |
|  | Vss | mL/kg | 15465.07 | 14608.75 | 20075.95 | 16716.59 ± 2940.63 |
|  | Vz | mL/kg | 21249.61 | 9605.48 | 17091.47 | 15982.19 ± 5900.79 |
|  | CL | mL/h/kg | 4772.80 | 3364.57 | 3711.33 | 3949.57 ± 733.72 |
|  | C_max_ | ng/mL | 853.98 | 733.88 | 573.21 | 720.36 ± 140.87 |

**Supplementary Table 7.** Summary of clinical observations

| **Detailed Clinical Observation Record of a Single Animal - Males** | | |
| --- | --- | --- |
|  |  |  |
| Groups |  |  |
| Animal ID | Symptomatic | Date |
| **Group 1：C-1 0 mg/kg**  **(solvent)** | |  |
| 101 | no abnormality | Day 1-Day 8 |
| 102 | no abnormality | Day 1-Day 8 |
| 103 | no abnormality | Day 1-Day 8 |
| **Group 2：C-1 500 mg/kg** | |  |
| 201 | no abnormality | Day 2-Day 8 |
|  | salivation | Day 1 |
| 202 | no abnormality | Day 1-4, Day 8 |
|  | Nasal discharge, red | Day 5, Day 6, Day 7 |
| 203 | no abnormality | Day 4, Day 6-8 |
|  | salivation | Day 1 |
|  | Nasal discharge, red | Day 2，Day 3，Day 5 |
| **Group 3：C-1 1000 mg/kg** | |  |
| 301 | no abnormality | Day 1-Day 8 |
| 302 | no abnormality | Day 1, Day 3-8 |
|  | Nasal discharge, red | Day 2 |
| 303 | no abnormality | Day 1-4, Day 7-8 |
|  | Nasal discharge, red | Day 5，Day 6 |
| **Group 4：C-1 2000 mg/kg** | |  |
| 401 | no abnormality | Day 1-Day 5 |
|  | Nasal discharge, red | Day 6，Day 7，Day 8 |
|  | Reduced activity | Day 8 |
| 402 | no abnormality | Day 1-Day 6 |
|  | Nasal discharge, red | Day 7，Day 8 |
|  | Reduced activity | Day 8 |
| 403 | no abnormality | Day 1-Day 6 |
|  | Nasal discharge, red | Day 7，Day 8 |
|  | Reduced activity | Day 8 |

| **Detailed Clinical Observation Record of a Single Animal - Females** | | |
| --- | --- | --- |
|  |  |  |
| Groups | Symptomatic |  |
| Animal ID |  | Date |
| **Group 1：C-1 0 mg/kg**  **(solvent)** | |  |
| 104 | no abnormality | Day 1-Day 8 |
| 105 | no abnormality | Day 1-Day 8 |
| 106 | no abnormality | Day 1-Day 8 |
| **Group 2：C-1 500 mg/kg** | |  |
| 204 | no abnormality | Day 1-Day 8 |
| 205 | no abnormality | Day 1, Day 5-Day 8 |
|  | Nasal discharge, red | Day2, Day3, Day4 |
| 206 | no abnormality | Day 1-Day 8 |
| **Group 3：C-1 1000 mg/kg** | |  |
| 304 | no abnormality | Day 1, Day 3-Day 8 |
|  | Nasal discharge, red | Day 2 |
| 305 | no abnormality | Day 2-Day 8 |
|  | salivation | Day 1 |
| 306 | no abnormality | Day 1-Day 8 |
| **Group 4：C-1 2000 mg/kg** | |  |
| 404 | no abnormality | Day 1-Day 2, Day 6-Day 8 |
|  | Nasal discharge, red | Day 3, Day 4, Day 5 |
| 405 | no abnormality | Day 1-Day 5, Day 8 |
|  | Nasal discharge, red | Day 6，Day 7 |
| 406 | no abnormality | Day 1-Day 2,Day 7-Day 8 |
|  | Nasal discharge, red | Day 3, Day 4, Day 5, Day 6 |

**Supplementary Table 8.** Animal weight data

| **Body weight data of single animals - kg - males** | | | | |
| --- | --- | --- | --- | --- |
|  |  |  |  |  |
| Groups | Date | | | |
| Animal ID |  | D3 | D7 | D8 |
| **Group 1：C-1 0 mg/kg**  **(solvent)** | |  |  |  |
| 101 | 267.3 | 281.2 | 309.7 | 314.8 |
| 102 | 285.4 | 302.3 | 328.4 | 336.5 |
| 103 | 293.6 | 308.5 | 344.4 | 353.8 |
| Mean | 282.1 | 297.3 | 327.5 | 335.0 |
| SD | 13.5 | 14.3 | 17.4 | 19.5 |
| **Group 2：C-1 500 mg/kg** | |  |  |  |
| 201 | 268.4 | 278.3 | 304.5 | 317.3 |
| 202 | 275.5 | 294.1 | 320.0 | 330.8 |
| 203 | 294.1 | 306.5 | 341.3 | 356.2 |
| Mean | 279.3 | 293.0 | 321.9 | 334.8 |
| SD | 13.3 | 14.1 | 18.5 | 19.8 |
| **Group 3：C-1 1000 mg/kg** | |  |  |  |
| 301 | 271.5 | 266.9 | 306.1 | 313.7 |
| 302 | 279.5 | 271.5 | 327.1 | 335.3 |
| 303 | 285.7 | 266.6 | 322.2 | 327.9 |
| Mean | 278.9 | 268.3 | 318.5 | 325.6 |
| SD | 7.1 | 2.7 | 11.0 | 11.0 |
| **Group 4：C-1 2000 mg/kg** | |  |  |  |
| 401 | 271.8 | 272.2 | 194.1 | 183.3 |
| 402 | 279.7 | 271.1 | 188.3 | 182.4 |
| 403 | 287.7 | 270.5 | 191.6 | 182.8 |
| Mean | 279.7 | 271.3 | 191.3 | 182.8 |
| SD | 8.0 | 0.9 | 2.9 | 0.5 |

| **Body weight data of single animals - kg - females** | | | | |
| --- | --- | --- | --- | --- |
|  |  |  |  |  |
| Groups | Date | | | |
| Animal ID |  | D3 | D7 | D8 |
| **Group 1：C-1 0 mg/kg**  **(solvent)** | |  |  |  |
| 104 | 216.9 | 219.4 | 233.8 | 240.9 |
| 105 | 231.4 | 234.2 | 244.2 | 240.2 |
| 106 | 248.4 | 236.4 | 274.2 | 277.3 |
| Mean | 232.2 | 230.0 | 250.7 | 252.8 |
| SD | 15.8 | 9.2 | 21.0 | 21.2 |
| **Group 2：C-1 500 mg/kg** | |  |  |  |
| 204 | 221.4 | 221.6 | 227.7 | 233.9 |
| 205 | 224.9 | 179.9 | 233.6 | 240.8 |
| 206 | 253.6 | 256.6 | 272.1 | 262.7 |
| Mean | 233.3 | 219.4 | 244.5 | 245.8 |
| SD | 17.7 | 38.4 | 24.1 | 15.0 |
| **Group 3：C-1 1000 mg/kg** | |  |  |  |
| 304 | 221.7 | 220.1 | 241.7 | 238.4 |
| 305 | 226.7 | 225.3 | 237.7 | 250.2 |
| 306 | 234.3 | 230.0 | 253.4 | 247.9 |
| Mean | 227.6 | 225.1 | 244.3 | 245.5 |
| SD | 6.3 | 5.0 | 8.2 | 6.3 |
| **Group 4：C-1 2000 mg/kg** | |  |  |  |
| 404 | 222.8 | 193.8 | 226.3 | 235.1 |
| 405 | 229.1 | 217.4 | 178.6 | 200.1 |
| 406 | 237.8 | 229.1 | 245.1 | 254.6 |
| Mean | 229.9 | 213.4 | 216.7 | 229.9 |
| SD | 7.5 | 18.0 | 34.3 | 27.6 |
